# Revealing the Molecular Mechanism of Gastric Cancer Marker Annexin A4 in Cancer Cell Proliferation Using Exon Arrays

**DOI:** 10.1371/journal.pone.0044615

**Published:** 2012-09-07

**Authors:** Li-Ling Lin, Hsuan-Cheng Huang, Hsueh-Fen Juan

**Affiliations:** 1 Department of Life Science, Institute of Molecular and Cellular Biology, National Taiwan University, Taipei, Taiwan; 2 Institute of Biomedical Informatics, Center for Systems and Synthetic Biology, National Yang-Ming University, Taipei, Taiwan; National Taiwan University, Taiwan

## Abstract

Gastric cancer is a malignant disease that arises from the gastric epithelium. A potential biomarker for gastric cancer is the protein annexin A4 (ANXA4), an intracellular Ca^2+^ sensor. ANXA4 is primarily found in epithelial cells, and is known to be involved in various biological processes, including apoptosis, cell cycling and anticoagulation. In respect to cancer, ANXA4-overexpression has been observed in cancers of various origins, including gastric tumors associated with *Helicobacter pylori* infection. *H. pylori* induces ANXA4 expression and intracellular [Ca^2+^]_i_ elevation, and is an important risk factor for carcinogenesis that results in gastric cancer. Despite this correlation, the role of ANXA4 in the progression of gastric tumors remains unclear. In this study, we have investigated whether ANXA4 can mediate the rate of cell growth and whether ANXA4 downstream signals are involved in tumorigenesis. After observing the rate of cell growth in real-time, we determined that ANXA4 promotes cell proliferation. The transcription gene profile of ANXA4-overexpressing cells was measured and analyzed by human exon arrays. From this transcriptional gene data, we show that overexpression of ANXA4 regulates genes that are known to be related to cancer, for example the activation of hyaluronan mediated motility receptor (RHAMM), AKT, and cyclin-dependent kinase 1 (CDK1) as well as the suppression of p21. The regulation of these genes further induces cancer cell proliferation. We also found Ca^2+^ could regulate the transmission of downstream signals by ANXA4. We suggest that ANXA4 triggers a signaling cascade, leading to increased epithelial cell proliferation, ultimately promoting carcinogenesis. These results might therefore provide a new insight for gastric cancer therapy, specifically through the modification of ANXA4 activity.

## Introduction

Gastric cancer is the second leading cause of cancer deaths worldwide and shows high prevalence in Asian populations. Although the incidence of gastric cancer is declining, the overall 5-year survival rate remains low [1]. Determining the most efficient gastric cancer therapies and developing early-stage diagnostic tools are important strategies in affecting clinical outcomes. The comprehensive investigation of the molecular mechanisms that underlie gastric carcinogenesis could provide assistance in developing useful therapeutic strategies for this disease.

*Helicobacter pylori* is a gastric pathogen and is the predominant etiological factor for gastric carcinogenesis. Approximately half of the world’s population is infected with *H. pylori*, and more than 60% of gastric cancer patients have a history of *H. pylori*-positivity [2], [3], [4]. Recent studies have showed that *H. pylori* can induce both the proliferation of gastric cancer cells and mucosal inflammatory responses [5], [6]. Thus, in order to investigate the molecular mechanisms underlying gastric carcinogenesis, it is necessary to investigate the role and mechanisms of *H. pylori* in gastric carcinogenesis.

Annexins are ubiquitously expressed in most organisms, including animals, plants, fungi and protists. It is associated with a variety of physiological functions [7]. Based on the structure of their conserved core domain, annexins are considered to be intracellular Ca^2+^ sensors and phospholipid binding proteins. They have been observed to stimulate membrane trafficking and vesicle aggregation in response to increased intracellular [Ca^2+^]_i_ [8], [9]. In humans, annexins have been observed to have a range of cellular functions that have been implied in cytoskeletal organization, exocytosis, endocytosis, ion channel regulation, inflammation, apoptosis, fibrinolysis and coagulation [8]. Annexins are also considered to be involved in cancer, diabetes and inflammation [10]. Recently, more and more studies have emerged that implicate the involvement of annexins in carcinogenesis, as well as promoting proliferation [11], [12], invasion [13] and metastasis [14], [15]. However, the relationship between all members of the annexins family with cancer has not been characterized.

Annexin A4 (ANXA4) is a member of the annexins family associated with the digestive system. It is prominently expressed in epithelial cells [16]. Recent studies have shown that ANXA4 is considered to be a potential gastric biomarker based on its identification in tissues of gastric cancer patients in proteomic studies. Up-regulation of ANXA4 is specifically found in *H. pylori*-infected tumor tissues [17], [18]. Additionally, many studies have reported a relationship between ANXA4 expression and cancer. This relationship has been reported in pancreatic adenocarcinoma [19], clear cell carcinoma of ovary [20], [21], renal carcinoma [22], colorectal carcinoma [23] and prostate cancer [24]. In renal cell carcinoma, up-regulation of ANXA4 is involved in dissemination of tumor cells, promoting cell migration [22]. In colorectal cancer patients, high expression of ANXA4 was associated with a low survival rate and was reported as a potential biomarker for tumor diagnosis [23]. Insofar as cellular function, ANXA4 could induce calcium signaling, anticoagulation and resistance to apoptosis [25], [26]. These events indicate that ANXA4 has a tumorigenic function by regulating cell growth rate.

In this study, we intended to elucidate the molecular mechanism by which ANXA4 induces carcinogenesis. To accomplish this, we monitored the growth rate of gastric cancer cells with different expression levels of ANXA4. We also evaluated the transcriptional expression profile of ANXA4-overexpressing cells and used exon arrays to analyze the downstream signaling of ANXA4. From these studies, we identified 9 cancer-related genes from ANXA4 downstream signals by using the Ingenuity Pathway Analysis (IPA) database. Furthermore, we demonstrated that ANXA4 regulates the activation of RHAMM, AKT, CDK1 and the suppression of p21, and therefore proposed an ANXA4-regulated cellular proliferation pathway.

## Results

### The Activation of ANXA4 Promotes Cell Proliferation

**Figure 1:**
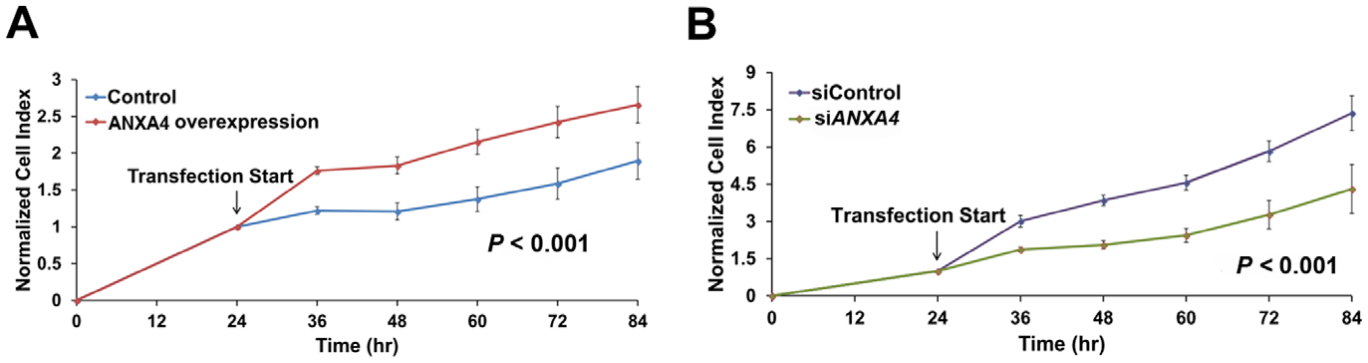
ANXA4 induces cell proliferation. To measure cell proliferation, AGS cells were cultured in a 16-well microtiter E-plate. After incubation for 24 h, the cell growth rate of (A) cells overexpressing ANXA4, and (B) cells containing *ANXA4*-specific siRNA were measured. It was observed that ANXA4 regulated the cell index in a time-dependent manner. (A and B) Data were normalized from measurements taken at 24 h, which was when transfection was initiated. The detection time from three independent experiments is represented as mean ± SD, n = 3. *P* values were calculated using the two-sample Kolmogorov-Smirnov test.

We have previously reported that ANXA4 is overexpressed in *H. pylori* infected gastric tumor tissue [17]. To further investigate the relationship between ANXA4 and carcinogenesis, we aimed to determine whether ANXA4 promotes cell proliferation. Cells were transfected with either full-length *ANXA4* cDNA to increase ANXA4 expression or specific siRNA intended to silence ANXA4 expression. After transfection, we monitored the growth rate of AGS gastric cancer cells using a real-time cell analysis (RTCA) system. Cell numbers were recorded as a cell index (CI) value. The growth curves of AGS cells were modified following transfection. The overexpression of ANXA4 significantly increased the cell growth rate in AGS cells compared with control cells (*P*<0.001; Figure 1A), whereas ANXA4 knockdown significantly decreased the growth rate (*P*<0.001; Figure 1B). These results suggest that ANXA4 has the potential to promote cell proliferation.

### ANXA4 Increases Expression of the Membrane Proteins, RHAMM and LAMP2

We also examined the difference in gene expression between ANXA4-overexpressing cells and control cells expressing an empty vector. We examined this using exon array analysis, which offers a more accurate view of gene-level expression by using four probes per exon, compared to the conventional 3′ arrays [27]. In Figure S1, the *X*-axis of the scatter plot displays the intensities of probe expression measured in one experiment and the *Y-*axis displays the intensities of probe expression measured in the other experiment. These results indicate a correlation between the results of both experiments and therefore indicate a consistency between our duplicate microarrays. The gene expression levels were calculated from the intensities measured via probe sets. Overall, the expression of 1,052 genes were found to be significantly different (*P*<0.05) between ANXA4-overexpressing cells and control cells.

**Figure 2:**
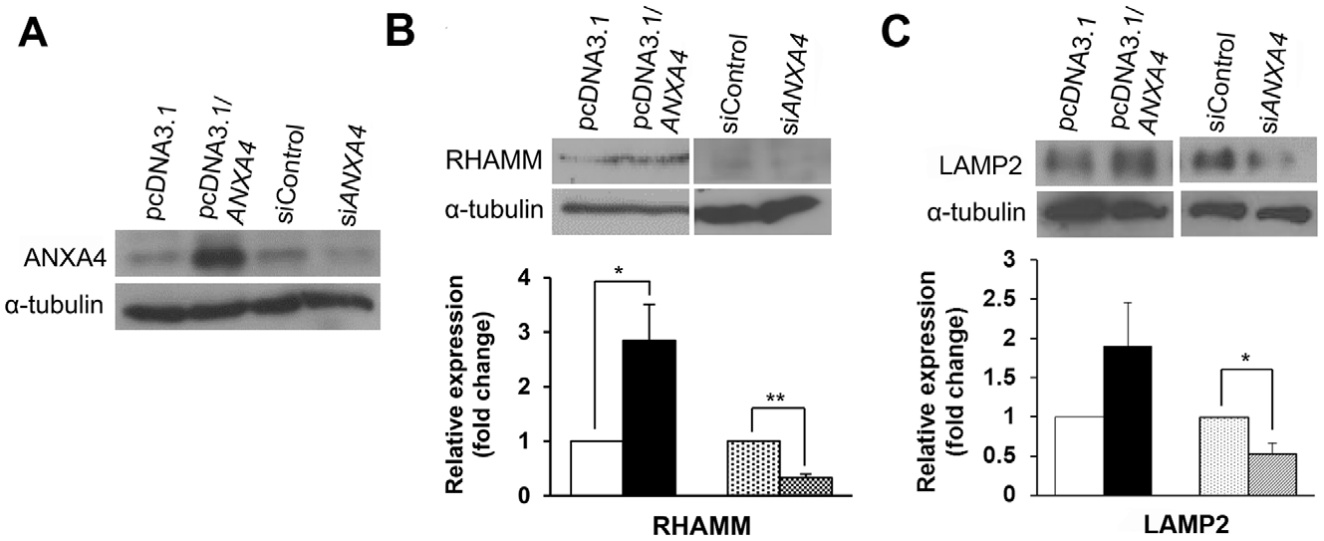
ANXA4 up-regulates RHAMM and LAMP2 expression. (A) AGS cells were transfected with either an empty vector (pcDNA3.1), full-length *ANXA4* (pcDNA3.1/*ANXA4*), control siRNA (siControl), or *ANXA4* siRNA (si*ANXA4*). (B–C) RHAMM and LAMP2 expressions were measured in ANXA4-overexpressing cells or cells containing *ANXA4*-specific siRNA by immunoblotting analysis. (B) The expression of RHAMM was significantly up-regulated (*P*<0.05) after overexpressing ANXA4 and significantly down-regulated (*P*<0.01) after silencing ANXA4 expression. (C) The expression of LAMP2 was up-regulated after overexpressing ANXA4 and significantly down-regulated (*P*<0.05) after silencing ANXA4 expression. Data are represented as mean ± SD, n = 3. *P<0.05 vs. control treatment values.

ANXA4 is a plasma membrane binding protein, so we propose that other plasma membrane proteins might be regulated by ANXA4 and work together to transduce its downstream signaling. To explore the signaling transduction regulated by ANXA4, we investigated plasma membrane proteins from exon array analysis. Forty-seven plasma membrane proteins were observed to have a ≥1.5-fold change in ANXA4-overexpressing cells (Table S1). Among these, hyaluronan-mediated motility receptor (*HMMR*) showed the largest increase in expression (2.4-fold; Table S1). Moreover, our previous study showed that the cell surface expression of lysosomal-associated membrane protein 2 (*LAMP2*), a lysosomal marker, is up-regulated by ANXA4 and is involved in exocytosis (Figure S2 and File S1). Here, transcriptional expression of the *LAMP2* also showed an increase in expression (1.8-fold; Table S1) when measured by exon array analysis. In this study, ANXA4 was overexpressed or silenced (Figure 2A) and then analyzed by immunoblotting to check the protein expression of RHAMM and LAMP2. ANXA4 overexpression increased the expression of RHAMM (Figure 2B) and LAMP2 (Figure 2C) and, consistent with this, the knockdown of ANXA4 expression decreased their levels of expression (Figure 2B and 2C).

### ANXA4 Overexpression Regulates Cancer-related Gene Expression

**Figure 3:**
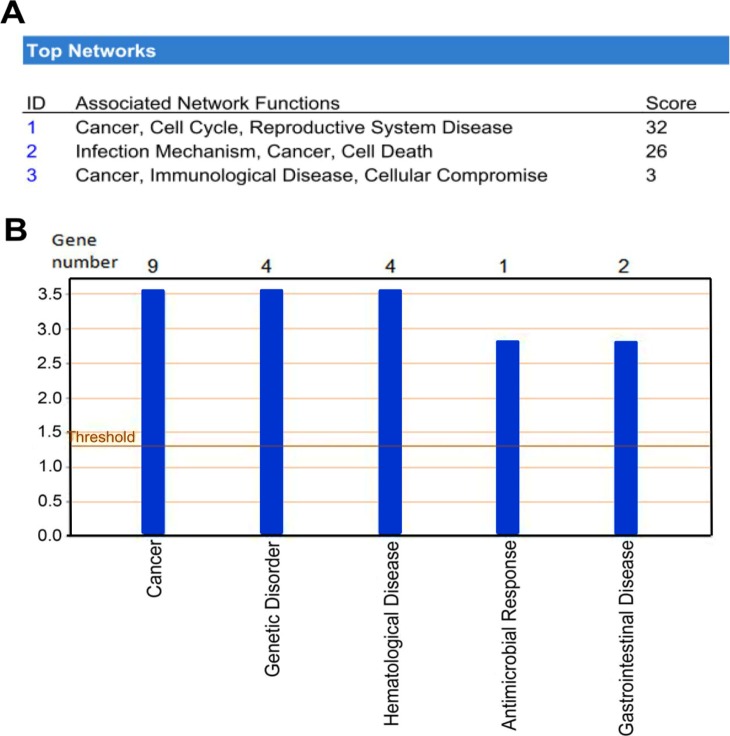
The significance of ANXA4-induced gene expression was analyzed using the Ingenuity Pathway Analysis (IPA) database. (A) 25 of the 42 candidate genes showed a significant difference in expression (a fold increase of ≥2 or a fold decrease of ≤0.5, *P*<0.05) and were categorized among the three top-ranked networks. (B) Genes were classified according to their documented/established roles in various disease states and disorders.

We used the Ingenuity Pathway Analysis (IPA) database to perform a gene function analysis of the exon array data and found that 25 of the 42 genes were eligible for network function analysis (≥2-fold differential expression, *t* test, *P*<0.05) (Table 1). Three functional networks were significantly associated with ANXA4-regulated genes. The most strongly associated network was *cancer, cell cycle, and reproductive system disease* (*P*<0.05; Figure 3A), and the top-ranked of diseases or disorders was *cancer* (*P*<0.05; Figure 3B). There were 9 genes classified as cancer-related genes in ANXA4-overexpressing cells including 7 genes that were up-regulated in our experiments. These genes are eukaryotic translation initiation factor 4E (*EIF4E*), succinate dehydrogenase complex, subunit C, integral membrane protein, 15 kDa (*SDHC*), cyclin-dependent kinase 1 (*CDK1*), deleted in lymphocytic leukemia 2 (*DLEU2*), chromatin modifying protein 5 (*CHMP5*), TIMELESS interacting protein (*TIPIN*), PDZ-binding kinase (*PBK*). Chorionic somatomammotropin hormone 1 (placental lactogen) (*CSH1*) and interferon, alpha 2 (*IFNA2*) were down-regulated by ANXA4. Based on our results, we propose that ANXA4 has a function in inducing cell proliferation. Moreover, *CDK1* and *PBK* (Figure 3B and Table 1) were considered to be involved in the ANXA4 proliferation-inducing model. Since CDK1 activation is associated with the cell proliferation in developing gastric MALT lymphoma [28]. The mutual activation of CDK1 and PBK has been previously reported [29]. In this study, the up-regulation of *CDK1* and *PBK* observed in ANXA4-overexpressing cells was validated by quantitative real-time polymerase chain reaction (qRT-PCR) analysis (Figure S3).

**Table 1:** Differentially expressed genes (two fold change) identified by microarray analysis and classified with the IPA database.

Gene Symbol	Gene Description	Fold Change	p-value
EIF4E* †	eukaryotic translation initiation factor 4E	4.7	0.022
FAM133B	family with sequence similarity 133, member B	3.5	0.026
SDHC* †	succinate dehydrogenase complex, subunit C, integral membrane protein, 15 kDa	3.2	0.006
MRPL41*	mitochondrial ribosomal protein L41	3.1	0.045
ACTR6*	ARP6 actin-related protein 6 homolog (yeast)	2.8	0.044
OR2M4	olfactory receptor, family 2, subfamily M, member 4	2.7	0.034
ZNF701*	zinc finger protein 701	2.5	0.010
GPN3*	GPN-loop GTPase 3	2.5	0.025
HIGD2A	HIG1 hypoxia inducible domain family, member 2A	2.4	0.017
RPS27L*	ribosomal protein S27-like	2.4	0.002
CDK1* †	cyclin-dependent kinase 1	2.4	0.041
DLEU2* †	deleted in lymphocytic leukemia 2 (non-protein coding)	2.4	0.041
CHMP5* †	charged multivesicular body protein 5	2.3	0.019
MRPL39	mitochondrial ribosomal protein L39	2.3	0.030
FCF1	FCF1 small subunit (SSU) processome component homolog (S. cerevisiae)	2.3	0.010
NDUFA4*	NADH dehydrogenase (ubiquinone) 1 alpha subcomplex, 4, 9 kDa	2.2	0.013
FBXO16*	F-box protein 16	2.2	0.036
LYPLA1*	lysophospholipase I	2.2	0.004
FSD1L	fibronectin type III and SPRY domain containing 1-like	2.2	0.042
TIPIN* †	TIMELESS interacting protein	2.1	0.019
	hypothetical LOC727817	2.1	0.018
ZDHHC20	zinc finger, DHHC-type containing 20	2.1	0.045
EXTL2*	exostoses (multiple)-like 2	2.1	0.044
COX16	COX16 cytochrome c oxidase assembly homolog (S. cerevisiae)	2.1	0.045
NMI*	N-myc (and STAT) interactor	2.1	0.040
PBK* †	PDZ-binding kinase	2.0	0.030
ANKRA2*	ankyrin repeat, family A (RFXANK-like), 2	2.0	0.036
PHOSPHO2	phosphatase, orphan 2	2.0	0.017
WBP5*	WW domain-binding protein 5	2.0	0.016
PSMA3*	proteasome (prosome, macropain) subunit, alpha type, 3	2.0	0.012
C17orf78	chromosome 17 open reading frame 78	0.5	0.030
C15orf2	chromosome 15 open reading frame 2	0.5	0.048
CSH1* †	chorionic somatomammotropin hormone 1 (placental lactogen)	0.5	0.037
C1QTNF9	C1q and tumor necrosis factor-related protein 9	0.5	0.008
IFNA2* †	interferon, alpha 2	0.5	0.048
KRTAP5-5	keratin-associated protein 5–5	0.5	0.024
TSPYL6	TSPY-like 6	0.4	0.037
SRP14*	signal recognition particle 14 kDa (homologous Alu RNA-binding protein)	0.4	0.020
OR13A1	olfactory receptor, family 13, subfamily A, member 1	0.4	0.030
OR10G7	olfactory receptor, family 10, subfamily G, member 7	0.4	0.048
PRH1*	proline-rich protein HaeIII subfamily 1	0.3	0.028
KRTAP4-12*	keratin-associated protein 4–12	0.3	0.036

* Gene eligible for network function analysis.

† Gene eligible for network function analysis and classified as a cancer-related gene.

### ANXA4 Regulates the Activation of AKT, CDK1 and the Suppression of p21

**Figure 4:**
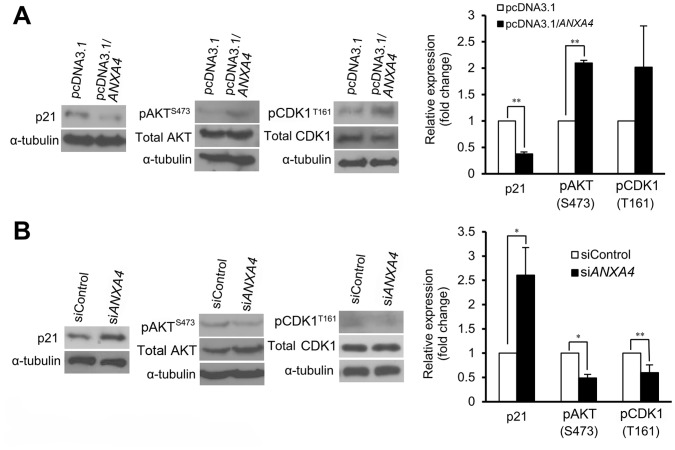
ANXA4 induces downstream signal transduction. (A*–*B) Protein levels of p21, phospho-AKT (Ser473) and phospho-CDK1 (Thr161) in AGS cells, as determined by immunoblotting analysis. (A) Cells were transfected with empty vector or full-length *ANXA4*. (B) Cells were transfected with siControl or si*ANXA4*. Representative data from three independent experiments are presented as mean ± SD. α-tubulin was used as an internal control. *P<0.05, **P<0.01 vs. control treatment values.

CDK1 activation, which is regulated by p21, inhibits G2/M phase arrest, thus promoting mitosis and cell proliferation [30]. It also has been reported that AKT blocks the activation of p21, causing its accumulation in the cytoplasm and consequently promoting cell proliferation [31]. Using immunoblot analysis, we observed that overexpression of ANXA4 increased the phosphorylation of serine 473 on AKT and threonine 161 on CDK1 and decreased the expression of p21 (Figure 4A). In addition to this, the knockdown of ANXA4 expression decreased phospho-AKT and phospho-CDK1 and increased the expression of p21 (Figure 4B). This data suggests that phospho-AKT, phospho-CDK1 and p21 are regulated by ANXA4 and are downstream signals of ANXA4.

### Ca^2+^ Mediates ANXA4 Downstream Signaling Transduction

**Figure 5:**
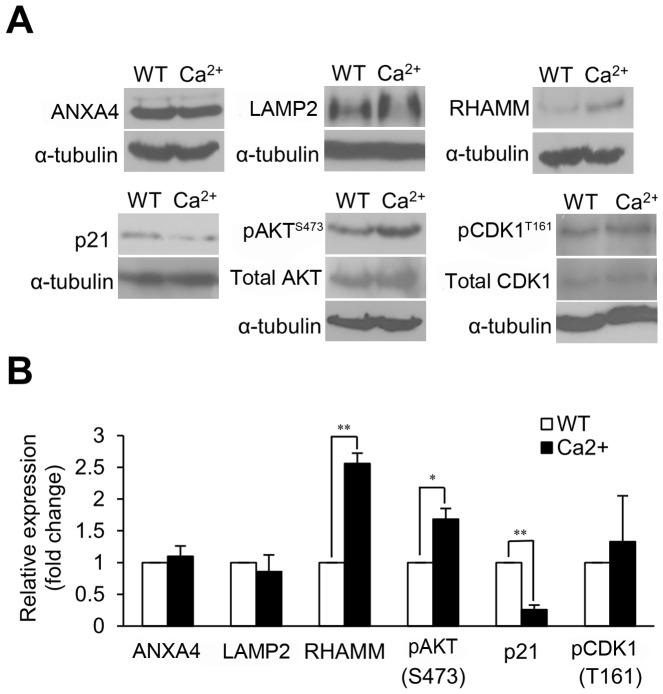
Ca^2+^ mediates the expression of RHAMM, phospho-AKT and p21. (A) Cells were treated with ionomycin to increase intracellular Ca^2+^ levels, and the expression levels of ANXA4, LAMP2, RHAMM, phospho-AKT (Ser473), p21, and phospho-CDK1 (Thr161) were showed by immunoblotting. (B) The histogram shows the related levels of (A). The relative expressions of RHAMM (*P*<0.01), phospho-AKT (Ser473) (*P*<0.05) and p21 (*P*<0.01) were significantly different. Data are taken from three independent experiments (mean ± SD). *P<0.05, **P<0.01 vs. control treatment values.

In carcinogenesis, Ca^2+^ is involved in causing signal transduction to mediate a wide variety of biological processes including invasion, proliferation, angiogenesis and metastasis [32]. It has been reported that annexins can mediate some physiological mechanisms in a Ca^2+^-dependent manner [9]. In previous studies, intracellular [Ca^2+^]_i_ elevation was induced by *H. pylori* infection, which, in turn, up-regulates ANXA4 expression [17], [33]. To determine whether an increase in intracellular [Ca^2+^]_i_ mediates the transmission of downstream signals from ANXA4, AGS cells were treated with ionomycin. Ionomycin is a Ca^2+^ ionophore and was added to the culture media in our AGS cell model to induce a sustained elevation of intracellular [Ca^2+^]_i_ [34]. The protein expressions of ANXA4, LAMP2, RHAMM, p21, phospho-AKT and phospho-CDK1 were measured by immunoblot analysis (Figure 5A). Similar to our observations with ANXA4 overexpression, increased [Ca^2+^]_i_ levels significantly up-regulated the expression of RHAMM (*P*<0.01) and phospho-AKT (Ser 473) (*P*<0.05) and significantly down-regulated the expression of p21 (*P*<0.01) (Figure 5B). CDK1 activation was slightly increased by [Ca^2+^]_i_ elevation. However, elevated [Ca^2+^]_i_ levels had no effect on the expression of ANXA4 and LAMP2. In our previous study, ANXA4 and LAMP2 can localize to plasma membrane after *H. pylori* infection with intracellular [Ca^2+^]_i_ elevation (Figure S4 and File S1). These results suggest that Ca^2+^ just changes intracellular location of ANXA4 and LAMP2, but not regulates their expression.

## Discussion

In cancer research, the identification of biomarkers and the subsequent clarification of their mechanistic relationships with tumorigenesis can contribute to the development of useful diagnostic tools and result in optimal therapeutic strategies. Recently, more and more studies have shown that the biomarker candidate proteins in the annexins family are potentially instrumental in the progression of various cancers; e.g., ANXA1 for clear cell renal cancer, ANXA2 for gastric cancer and colorectal cancer, ANXA4 for colorectal cancer, ANXA8 for breast cancer, ANXA10 for hepatocellular cancer, ANXA11 for ovarian cancer and colorectal cancer [35]. ANXA4, a member of the annexins family, has been observed to be overexpressed in gastric tumor tissues and is also associated with the gastric cancer-related *H. pylori* infection [17]. In addition, another annexins family member, ANXA2, has also been observed to be overexpressed in gastric cancer and is related to poor clinical outcome, making it a potential prognostic factor [36]. Taken together, these findings suggest the involvement of ANXA4 in tumorigenesis; however, its exact mechanism in the process remains unclear. In order to further evaluate the cellular function of ANXA4 in the progression of gastric cancer, we explored the link between the two and subsequently demonstrated that ANXA4 may regulate cancer-related genes and propagate the path to cell proliferation.

**Figure 6:**
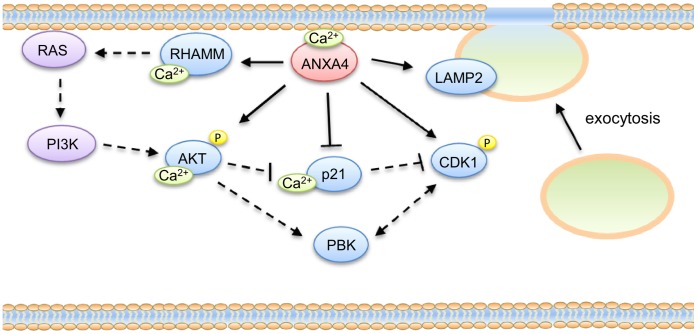
Schematic representation of the molecular mechanism is induced by ANXA4. ANXA4 binds to the plasma membrane in a Ca^2+^-dependent manner and induces downstream signaling transduction. ANXA4 up-regulates LAMP2, a lysosomal marker involved in exocytosis, and RHAMM. Previous reports have showed that RHAMM activates RAS and PI3K, which subsequently leads to the induction of AKT. ANXA4 up-regulates AKT and CDK1 activation, PBK gene expression and down-regulates p21. Ca^2+^ also up-regulates RHAMM and phospho-AKT, and down-regulates p21. This signal cascade might eventually lead to cell hyperproliferation. Solid lines with arrows and blue circles indicate confirmed regulation; dashed lines with arrows and purple circles indicate references or unconfirmed interactions.

In the present study, we found that carcinogenesis-associated proteins such as RHAMM, AKT, p21, PBK, and CDK1 are regulated by the overexpression of ANXA4. A schematic representation of ANXA4-induced downstream signals related to cell proliferation is shown in Figure 6. *HMMR* (RHAMM) is an oncogene that is overexpressed in several cancers, including gastric cancer, and has been implicated in many cellular processes, such as cell signaling, cell proliferation, and tumorigenesis [37], [38].

It has been reported that RHAMM induces the RAS signaling cascade and activates AKT [39]. The RAS signaling cascade transduces downstream signals by activating phospho-AKT through phosphoinositide 3-kinases. This activation of AKT has also been reported as a marker for gastric cancer progression [40]. We have previously reported that *H. pylori* infection is associated with ANXA4 overexpression [17]. *H. pylori* can deliver the cytotoxin associated gene A (CagA) into host cells, resulting in AKT activation, and thus promoting cell proliferation [41]. In terms of cell survival, AKT suppresses apoptosis by stimulating NF-κB [42]. Recent studies have also shown that ANXA4 interacts with p105 (the NF-κB p50 precursor protein) and suppresses the transcriptional activity of NF-κB to induce an anti-apoptotic effect [25]. Taken together, these observations indicate that ANXA4 plays an important role in cell survival and cell growth.

CDK1-cyclin B1 complexes regulate the cell cycle G2/M phase and have also been implicated in promoting tumorigenesis [43]. A recent study revealed that increased expression of CDK1 is associated with the progression from *H. pylori*-associated gastritis to mucosa-associated lymphoid tissue lymphoma [28]. In this study, CDK1 activation was up-regulated by ANXA4. These events suggest that ANXA4 could mediate the downstream signal pathway, leading to tumorigenesis in gastric cancer patients with a *H. pylori* infection.

In addition to its involvement in the progression of cancer, ANXA4 has also been linked to acquired chemoresistance to anticancer drugs [44], [45], [46]. In a paclitaxel-resistant cell line (H460/T800), ANXA4 expression is increased and localized in the nucleus [44]. In clear cell carcinoma of the ovary and mesothelioma cells, ANXA4 expression is elevated and associated with resistance to treatment with carboplatin, and is considered to be a biomarker for susceptibility to cisplatin [45], [46]. Moreover, ANXA4 is an intracellular Ca^2+^ sensor, and Ca^2+^ plays an important role in neurotoxicity and heart failure [47], [48]. Recent studies have shown that the increased amount of ANXA4 is not only associated with cancer but also Alzheimer’s disease, heart failure and cell lesion caused by ethanol [49], [50], [51].

Ca^2+^ messenger system is also required for cell proliferation process and can regulate cell cycle [52], [53]. It has been reported that Ca^2+^ can activate AKT pathway to promote cell survival [54]. In this study, we found that the expression of RHAMM, phospho-AKT and the suppression of p21 were significantly increased by [Ca^2+^]_i_ elevation (Figure 5). *H. pylori* infection is associated with ANXA4 overexpression and intracellular [Ca^2+^]_i_ elevation (Figure S4 and File S1) [17], [33]. These results indicate that *H. pylori* infection might induce some downstream signaling of ANXA4 by stimulating intracellular [Ca^2+^]_i_ elevation. Nevertheless the detail mechanism in the process might be complex and remains unclear. These could provide elucidation of gastric tumorigenesis process underlying *H. pylori* stimulation. Taken together, this evidence suggests that Ca^2+^ might assist ANXA4 to transduce signaling and promote tumorigenesis in gastric patients with *H. pylori* infection.

In conclusion, our results show that the silencing of ANXA4 decreases epithelial cell proliferation, while its overexpression increases proliferation. Furthermore, ANXA4 induces downstream signals that promote cell growth. We hypothesize that these downstream signals and activation of host cell division may be pathogenic events in *H. pylori*-induced carcinogenesis. In current clinical therapy, AKT, p21 and CDK1 have been used as an anticancer drug target. AKT inhibitor, Perifosine, is an oral anti-cancer agent and has anti-proliferation activity in several tumor models [55], [56], [57]. Paclitaxel (Taxol) and vincristine can induce and increase p21 expression to decrease G2/M arrest and block cell proliferation [58], [59], [60], [61]. Flavopiridol is an inhibitor of several CDKs including CDK1 to induce apoptosis and anti-angiogenesis [62]. These studies indicate that the elevation of ANXA4 in patients could be considered as a drug target for gastric cancer therapy. Using multiple drugs in combination might provide more effective treatment for blocking signals in the pathway. Taken together, this study could provide new insights into the development of therapeutic strategies for gastric cancer.

## Materials and Methods

### Cell Lines and Culture Conditions

Human stomach adenocarcinoma AGS cells (CRL-1739, ATCC) were grown in 90% RPMI 1640 medium (Biological Industries, Beth-Haemek, Israel) that was supplemented with 1% penicillin/streptomycin and 10% fetal bovine serum (Biological Industries, Beth-Haemek, Israel). Cells were cultured at 37°C in a controlled humidified atmosphere in an incubator containing 5% CO_2_.

### Plasmids and Transfections

Full-length *ANXA4* was amplified by PCR using the primer pair *ANXA4*-F (5′ atataagcttgccaccatggccatggcaaccaaa 3′) and *ANXA4*-R (5′ gcgcgggaattcttaatcatctcctccaca 3′), and the amplification product was inserted into the HindIII/EcoRI sites of pcDNA 3.1(+) (Invitrogen, Carlsbad, CA). *ANXA4*-specific siRNA and negative control Stealth siRNA (Stealth RNAi™) were purchased from Invitrogen (Carlsbad, CA, USA). Cells were cultured in six-well plates or on coated cover slips for 24 h. Cells were then transiently transfected with pcDNA 3.1(+)/*ANXA4* (8 µg for a six-well plate; 0.4 µg/mL for a 96-well E-plate) or *ANXA4* siRNA (100 pmol for a six-well plate; 10 pmol for a 96-well E-plate) using Lipofectamine 2000 (Invitrogen) according to the manufacturer’s instructions. The efficiency of expression vector and siRNA transfection was analyzed by immunoblot. After transfection for 48 h, the differential expression of proteins and genes was detected.

### Antibodies

The mouse monoclonal antibodies used in this study were as follows: CDK1 p34 (sc-51578) from Santa Cruz Biotechnology (Santa Cruz, CA, USA); LAMP2 (ab25631) and RHAMM (ab67003) from Abcam (Cambridge, UK); and α-tubulin (T5168) from Sigma (Dorset, UK). The rabbit polyclonal antibodies were as follows: ANXA4 (sc-28827), p21 (sc-397), and pCDK1 p34 (Thr 161; sc-101654) from Santa Cruz Biotechnology; and AKT (9272) and pAKT (Ser473; 9271S) from Cell Signaling Technology (Beverly, MA, USA).

### Immunoblotting

Cell lysates were prepared from AGS cells that were transiently transfected with the expression vectors pcDNA 3.1(+)/*ANXA4* (8 µg) or *ANXA4* siRNA (100 pmol). To determine the differences in protein expression under conditions of high intracellular [Ca^2+^]_i_, ionomycin (5 µM) was added to the cells for 1 h. To study the effects of inhibition of Akt phosphorylation, the cells were starved for 1.5 h following a 48-h transfection and were treated with 5 µM of the AKT inhibitor VIII (Merck KGaA, Darmstadt, Germany) for 1 h. Samples were separated by 10% SDS-PAGE and then transferred onto polyvinylidene difluoride (PVDF) membranes (Millipore, Billerica, MA, USA). After blocking in 5% nonfat milk and tris-buffered saline (TBS) containing 0.1% Tween 20 (JT Baker, Phillipsburg, NJ, USA) for 1 h at RT with gentle rocking, the following primary antibodies were applied: anti-ANXA4 (1∶1000), anti-p21 (1∶500), anti-AKT (1∶500), anti-pAKT (Ser473; 1∶500), anti-CDK1 (1∶1000), anti-pCDK1 (Thr 161; 1∶500), and anti-RHAMM antibody (1∶400). Membranes were incubated with secondary goat anti-mouse conjugated IgG antibodies (Sigma) or goat anti-rabbit conjugated IgG (Rockland, Gilbertsville, PA), respectively. α-tubulin antibody (1∶4000) was used as an internal control. Immunoblots were developed using enhanced chemiluminiscence (ECL) detection kit (Millipore) and visualized on X-ray films. The intensity of the observed bands was normalized to the intensity of the α-tubulin band. Densitometric analysis was performed using Kodak 1-D Image Analysis software version 3.6 (Eastman Kodak, London, UK).

### Exon Array Hybridization and Analysis

To study the downstream genes of ANXA4, we compared the gene expression profiles between the cells transfected with pcDNA3.1 (+)/*ANXA4* and control cells transfected with an empty vector using an exon array. Total cellular RNA was extracted using TRIzol® Reagent (Invitrogen), and RNA purity was confirmed by spectrophotometry (A260/A280 ratio) as well as by capillary electrophoresis (Agilent 2100 Bioanalyzer, Agilent Technologies, Palo Alto, CA, USA). RNA processing and hybridization were performed using the Affymetrix Human Exon 1.0 ST arrays (Affymetrix, Santa Clara, CA, USA) according to the manufacturer’s protocol. Each array has 28,869 well-annotated genes with 764,885 different probes. The array contains approximately 26 probes for each gene. Affymetrix probe sets information are displayed on NetAffx Web site (http://www.affymetrix.com) [63]. Microarray analysis (n = 2 per group) was performed using the Partek Genomics Suite version 6.5 (Partek Inc., St Louis, MO, USA). The raw data (CEL files) were normalized using the robust multichip averaging (RMA) algorithm and analyzed using *t* tests. Analysis of the function and biological mechanism of the differentially expressed genes was performed using the Ingenuity Pathway Analysis (IPA) software version 7.5; a score of 3 or above was considered statistically significant (*P*<0.01) to annotate the information. We have submitted the array data to the GEO database, and the series record number is GSE33620.

### Quantitative Real-time PCR (qRT-PCR)

The exon array data obtained using Partek software and the IPA database was confirmed by qRT-PCR. RNA was isolated from AGS cells using TRIzol® Reagent (Invitrogen) and the RNeasy® Mini Kit (Qiagen, Hilden, Germany) according to the manufacturer’s instructions. First-strand cDNA was synthesized from total mRNA using a reverse transcription kit (Invitrogen, Carlsbad, CA). Primers (Table S2) were designed using DNAStar software (DNAStar, Inc., Madison, WI, USA) and PrimerBank (http://pga.mgh.harvard.edu/primerbank). Gene expression was measured using a Bio-Rad iQ5 real-time PCR detection system with an SYBR Green Supermix (Bio-Rad Laboratories, Hercules, CA, USA), and was normalized to GAPDH expression.

### Cell Proliferation Assay

AGS cells were loaded in each well of a 16-well microtiter E-plate. Each well contained microelectronic sensor arrays at the base to detect the cell index (CI). For transfection experiments, after incubation for 24 h, AGS cells were transfected with expression vectors or siRNAs for 6 h and monitored for a total of 84 h. The E-plate was placed in the Real-Time Cell Analyzer (RTCA) system and incubated in an incubator containing 5% CO_2_ at 37°C. The level of cell proliferation was represented as CI, which was based on the electrical impedance measured using the xCELLigence system (Roche, Mannheim, Germany).

### Statistical Analysis

Data were expressed as mean ± standard deviation (SD). Difference between independent groups was analyzed using a two-tailed Student’s *t* test. Data obtained from the cell proliferation assay were analyzed using the two-sample Kolmogorov-Smirnov test. A *P* value of less than 0.05 indicated statistical significance.

## Supporting Information

Figure S1 **Scatter plot of the probe intensities in the repeated exon array experiments.** The probe intensities of two repeated experiments were presented separately on an *X*-axis and *Y*-axis. Each probe was represented by a single dot in the scatter plot. These results showed the consistency in our duplicate exon array experiments.
(TIF)

Figure S2 **ANXA4 participates in plasma membrane repair by recruiting exocytotic membrane.** Representative flow cytometric analyses demonstrated the presence of LAMP2 in *H. pylori*-infected cells. (A) ANXA4-overexpressing cells were compared with (B) *ANXA4*-silenced cells. The results indicate that ANXA4 promotes LAMP2 expression on the surface of *H. pylori*-infected cells. ANXA4 overexpression, Over-ANXA4; Control siRNA, siControl; *ANXA4* siRNA, si*ANXA4*.
(TIF)

Figure S3 **ANXA4 induces downstream signal transduction.** A qRT-PCR assay of ANXA4-overexpressing AGS cells (black boxes) was performed to confirm the data obtained from exon arrays (gray boxes). The relative mRNA levels of *CDK1* and *PBK* were measured and normalized to *GAPDH* mRNA levels.
(TIF)

Figure S4 **Intracellular Ca^2+^ elevation, ANXA4 and LAMP2 localization upon** ***H. pylori*** **infection.** (A) *H. pylori*-infected AGS cells were loaded with Fluo-3/AM to monitor intracellular Ca^2+^ levels by flow cytometry. (B) Dynamic localization of ANXA4 in the living cell. Real-time fluorescence images showing localization of EGFP-ANXA4 in *H. pylori*-infected AGS and SC-M1 cells (yellow arrow) stained with Hoechst 33258. (C) LAMP2 fluorescence on the surface of *H. pylori*-infected AGS cells was more enhanced than on the surface of non-infected cells.
(TIF)

Table S1 **ANXA4-upregulated genes (fold-change ≥1.5) in AGS cells based on an exon array classified as plasma membrane proteins with the IPA database.**
(PDF)

Table S2 **List of primer sequences used for qRT-PCR.**
(PDF)

File S1 **Supplementary Materials and Methods.**
(PDF)
