# Supplementary figures and images for: Revealing the Molecular Mechanism of Gastric Cancer Marker Annexin A4 in Cancer Cell Proliferation Using Exon Arrays
Source: PLoS One. 2012 Sep 7;7(9):e44615. doi: 10.1371/journal.pone.0044615 (PMC3436854; doi:10.1371/journal.pone.0044615)

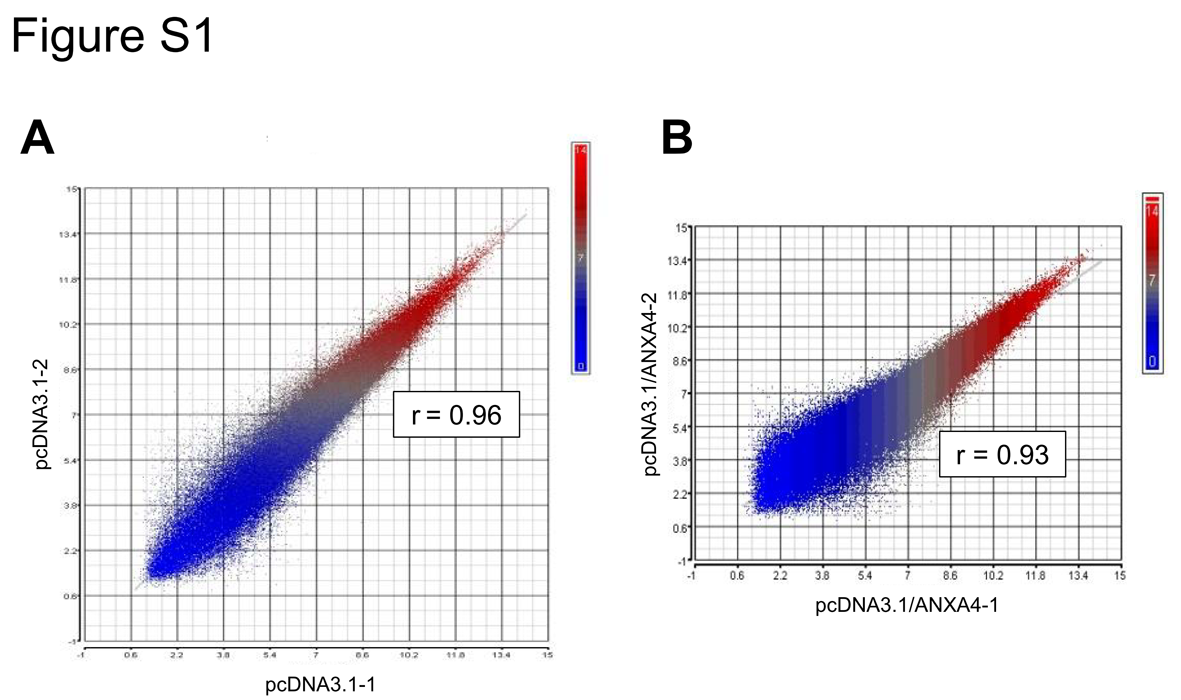

Supplement: Figure S1 — Scatter plot of the probe intensities in the repeated exon array experiments. The probe intensities of two repeated experiments were presented separately on an X-axis and Y-axis. Each probe was represented by a single dot in the scatter plot. These results showed the consistency in our duplicate exon array experiments. (TIF) [file pone.0044615.s001.tif]

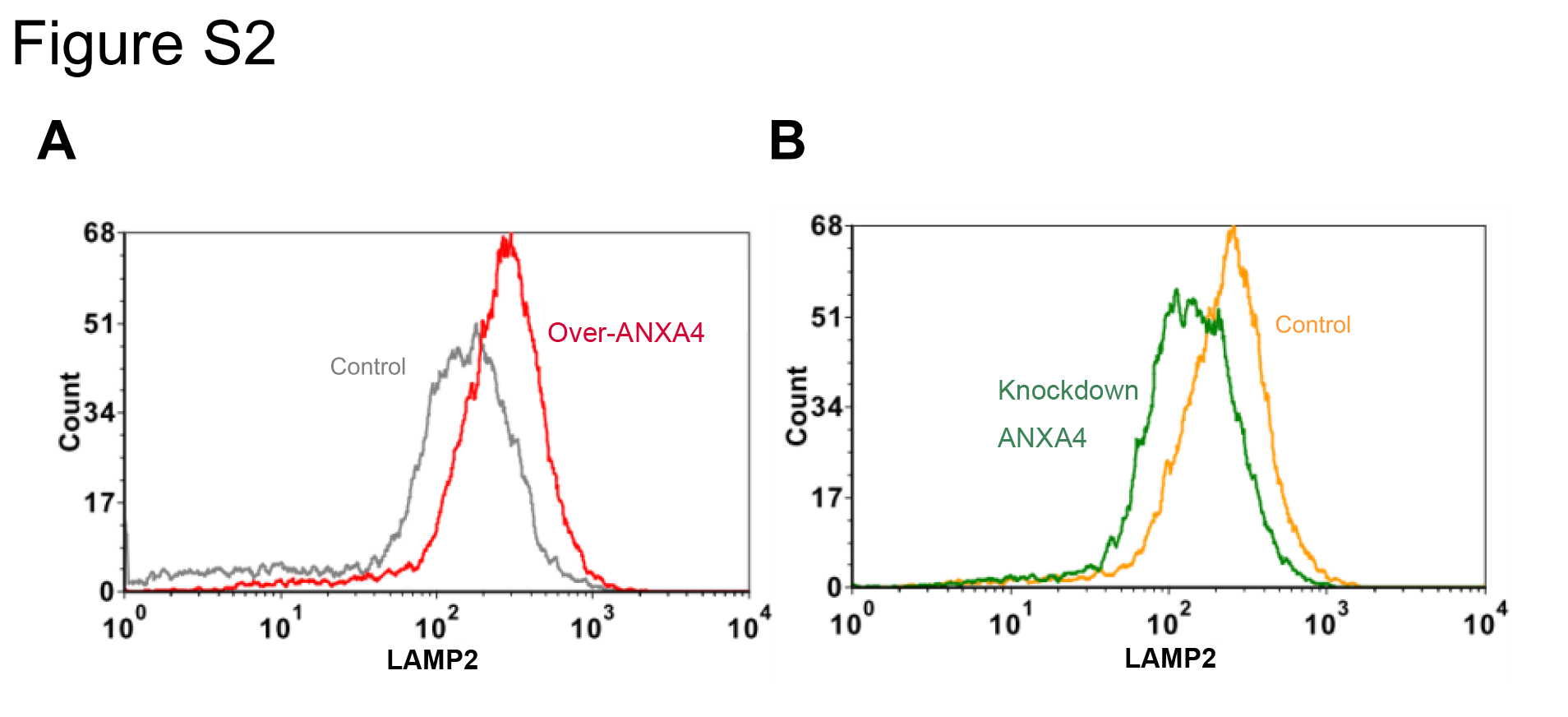

Supplement: Figure S2 — ANXA4 participates in plasma membrane repair by recruiting exocytotic membrane. Representative flow cytometric analyses demonstrated the presence of LAMP2 in H. pylori-infected cells. (A) ANXA4-overexpressing cells were compared with (B) ANXA4-silenced cells. The results indicate that ANXA4 promotes LAMP2 expression on the surface of H. pylori-infected cells. ANXA4 overexpression, Over-ANXA4; Control siRNA, siControl; ANXA4 siRNA, siANXA4. (TIF) [file pone.0044615.s002.tif]

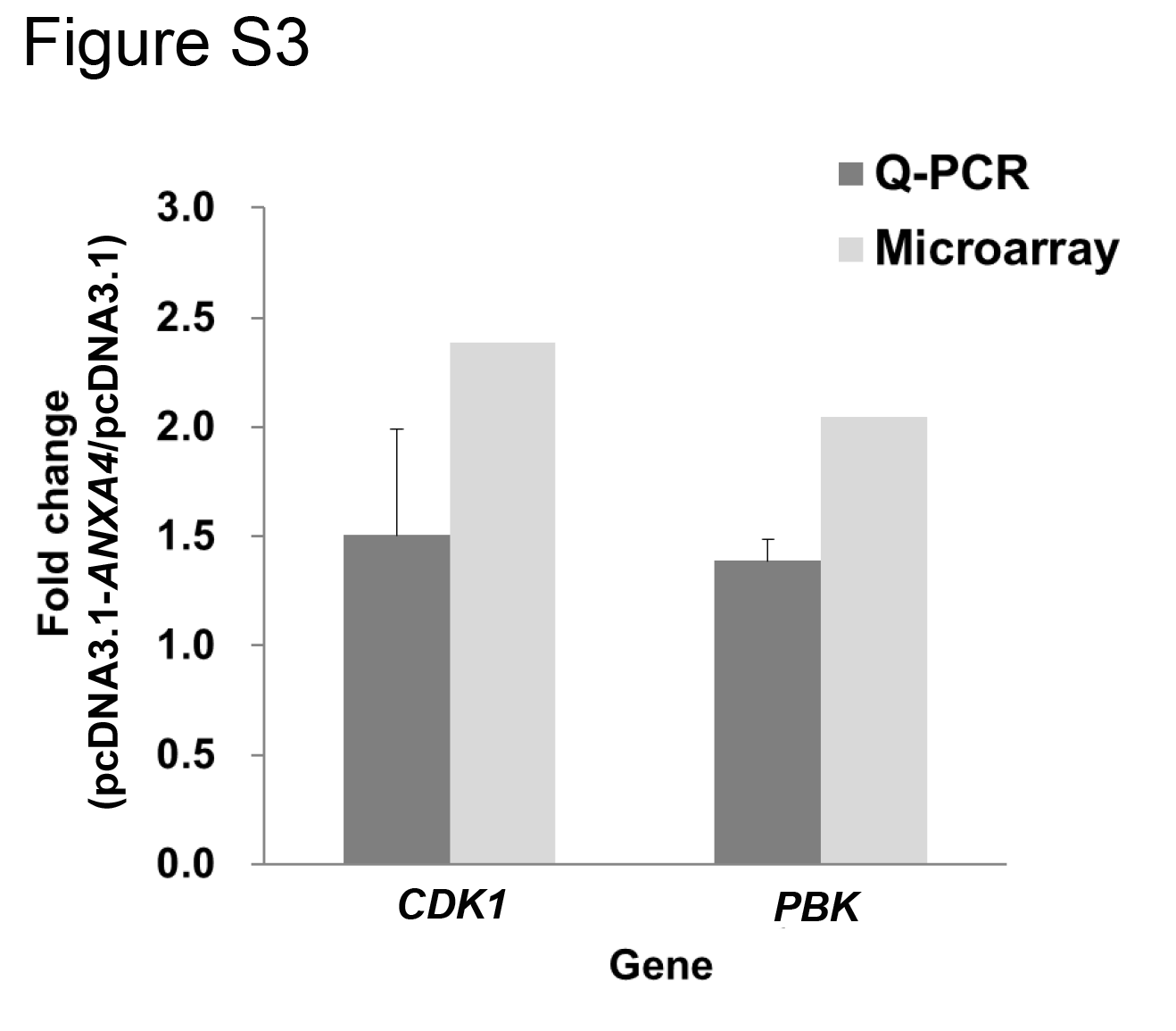

Supplement: Figure S3 — ANXA4 induces downstream signal transduction. A qRT-PCR assay of ANXA4-overexpressing AGS cells (black boxes) was performed to confirm the data obtained from exon arrays (gray boxes). The relative mRNA levels of CDK1 and PBK were measured and normalized to GAPDH mRNA levels. (TIF) [file pone.0044615.s003.tif]

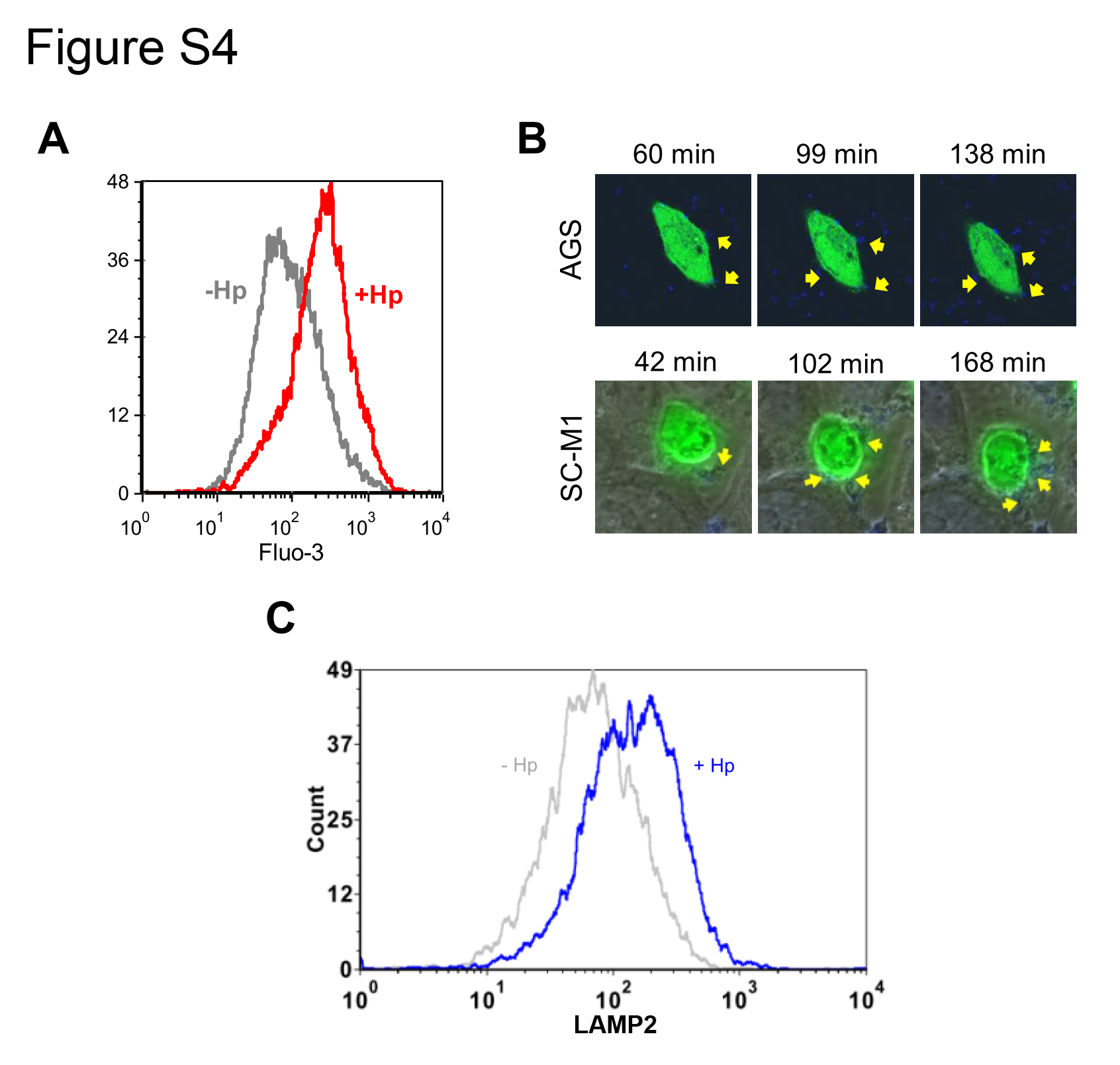

Supplement: Figure S4 — Intracellular Ca2+ elevation, ANXA4 and LAMP2 localization upon H. pylori infection. (A) H. pylori-infected AGS cells were loaded with Fluo-3/AM to monitor intracellular Ca2+ levels by flow cytometry. (B) Dynamic localization of ANXA4 in the living cell. Real-time fluorescence images showing localization of EGFP-ANXA4 in H. pylori-infected AGS and SC-M1 cells (yellow arrow) stained with Hoechst 33258. (C) LAMP2 fluorescence on the surface of H. pylori-infected AGS cells was more enhanced than on the surface of non-infected cells. (TIF) [file pone.0044615.s004.tif]
