# Supplementary material for: Revealing the Molecular Mechanism of Gastric Cancer Marker Annexin A4 in Cancer Cell Proliferation Using Exon Arrays
Source: PLoS One. 2012 Sep 7;7(9):e44615. doi: 10.1371/journal.pone.0044615 (PMC3436854; doi:10.1371/journal.pone.0044615)
Supplement: Table S1 — ANXA4-upregulated genes (fold-change ≥1.5) in AGS cells based on an exon array classified as plasma membrane proteins with the IPA database. (PDF) [file pone.0044615.s005.pdf]

Table S1 AnxA4-upregulated genes (fold-change ≥1.5) in AGS cells based on an exon array classified as plasma membrane proteins with the IPA database.

Gene Symbol	Description	Fold Change	Type
HMMR	hyaluronan-mediated motility receptor (RHAMM)	2.4	other
OR4P4	olfactory receptor, family 4, subfamily P, member 4	2.3	G-protein coupled receptor
OR5R1	olfactory receptor, family 5, subfamily R, member 1	2.2	G-protein coupled receptor
CXADR	coxsackie virus and adenovirus receptor	2.2	transmembrane receptor
OR7A17	olfactory receptor, family 7, subfamily A, member 17	2.1	G-protein coupled receptor
SLCO1B3	solute carrier organic anion transporter family, member 1B3	2	transporter
CHRM2	cholinergic receptor, muscarinic 2	2	G-protein coupled receptor
OR1J2	olfactory receptor, family 1, subfamily J, member 2	1.9	G-protein coupled receptor
STXBP3	syntaxin binding protein 3	1.8	transporter
LAMP2	lysosomal-associated membrane protein 2	1.8	enzyme
CALM2	calmodulin 2 (phosphorylase kinase, delta)	1.8	other
LMBR1	limb region 1 homolog (mouse)	1.8	transmembrane receptor
OR4A15	olfactory receptor, family 4, subfamily A, member 15	1.8	G-protein coupled receptor
OR2B2	olfactory receptor, family 2, subfamily B, member 2	1.7	G-protein coupled receptor
SSX2IP	synovial sarcoma, X breakpoint 2 interacting protein	1.7	other
CHRNA5	cholinergic receptor, nicotinic, alpha 5 (neuronal)	1.7	transmembrane receptor
GJA1	gap junction protein, alpha 1, 43kDa	1.7	transporter
STEAP1	six transmembrane epithelial antigen of the prostate 1	1.7	transporter

Table S1. Continued.

Gene Symbol	Description	Fold Change	Type
ITFG1	integrin alpha FG-GAP repeat containing 1	1.7	other
OR5T3	olfactory receptor, family 5, subfamily T, member 3	1.7	G-protein coupled receptor
TMEM67	transmembrane protein 67	1.7	other
CD24	CD24 molecule	1.6	other
IFNGR1	interferon gamma receptor 1	1.6	transmembrane receptor
OR4C3	olfactory receptor, family 4, subfamily C, member 3	1.6	G-protein coupled receptor
AQP9	aquaporin 9	1.6	transporter
STX3	syntaxin 3	1.6	transporter
ANXA7	annexin A7	1.6	ion channel
TMEM50B	transmembrane protein 50B	1.6	other
IFNAR1	interferon (alpha, beta and omega) receptor 1	1.6	transmembrane receptor
OR2M2	olfactory receptor, family 2, subfamily M, member 2	1.6	G-protein coupled receptor
SPAM1	sperm adhesion molecule 1 (PH-20 hyaluronidase, zona pellucida binding)	1.6	enzyme
LPAR6	lysophosphatidic acid receptor 6	1.6	G-protein coupled receptor
TAS2R14	taste receptor, type 2, member 14	1.6	G-protein coupled receptor
GPR65	G protein-coupled receptor 65	1.5	G-protein coupled receptor
SLC16A4	solute carrier family 16, member 4 (monocarboxylic acid transporter 5)	1.5	transporter
CLDN12	claudin 12	1.5	other
CTNNAL1	catenin (cadherin-associated protein), alpha-like 1	1.5	other
PSMG1	proteasome (prosome, macropain) assembly chaperone 1	1.5	other
CD58	CD58 molecule	1.5	other
SLC9A6	solute carrier family 9 (sodium/hydrogen exchanger), member 6	1.5	transporter

Table S1. Continued.

Gene Symbol	Description	Fold Change	Type
OR4X2	olfactory receptor, family 4, subfamily X, member 2	1.5	G-protein coupled receptor
DEGS1	delta(4)-desaturase, sphingolipid 1	1.5	enzyme
OR8K5	olfactory receptor, family 8, subfamily K, member 5	1.5	G-protein coupled receptor
HLA-DRB5	major histocompatibility complex, class II, DR beta 5	1.5	transmembrane receptor
GPR82	G protein-coupled receptor 82	1.5	G-protein coupled receptor
TSPAN8	tetraspanin 8	1.5	other
ANXA1	annexin A1	1.5	other
