# Supplementary material for: Revealing the Molecular Mechanism of Gastric Cancer Marker Annexin A4 in Cancer Cell Proliferation Using Exon Arrays
Source: PLoS One. 2012 Sep 7;7(9):e44615. doi: 10.1371/journal.pone.0044615 (PMC3436854; doi:10.1371/journal.pone.0044615)
Supplement: Table S2 — List of primer sequences used for qRT-PCR. (PDF) [file pone.0044615.s006.pdf]

Table S2. A list of primer sequences used for qRT-PCR

Primer	Sequence (5’→3’)
ANXA4-F	accatcggcagggacttgata
ANXA4-R	cagctcttgcacgtcatacag
CDK1-F	acacaaaactacaggtcaagtgg
CDK1-R	agagtgacaaaacacaatcccc
PBK-F	ggcctctccgtttatgcagaa
PBK-R	agcacgataaccaacaatgtttg
