# Supplementary material for: Revealing the Molecular Mechanism of Gastric Cancer Marker Annexin A4 in Cancer Cell Proliferation Using Exon Arrays
Source: PLoS One. 2012 Sep 7;7(9):e44615. doi: 10.1371/journal.pone.0044615 (PMC3436854; doi:10.1371/journal.pone.0044615)
Supplement: File S1 — Supplementary Materials and Methods. (PDF) [file pone.0044615.s007.pdf]

Supporting Information

Materials and Methods

Bacterial strains and culture conditions

The *H. pylori* (NTUH-GC05) strain (*vacA*+/*cagA*+) from the stomach of a male gastric cancer patient at the National Taiwan University Hospital was obtained in 1991, as described elsewhere [1]. *H. pylori* was grown on Columbia blood agar base (BD Difco) containing 5% sheep blood and incubated for 2–3 days in microaerophilic conditions (5% O_2, 10% CO_2, 85% N_2) at 37℃.

Plasmids and transfections

Full-length *ANXA4* was amplified by PCR using the primer pair *ANXA4*-F and *ANXA4*-R, and the amplification product was inserted into the HindIII/EcoRI sites of pcDNA3.1(+) (Invitrogen). For immunofluorescence analysis, *ANXA4* was amplified by PCR using the primer pair *ANXA4*-F2 and *ANXA4*-R2, and the amplification product was inserted into the HindIII/pst I sites of pEGFP-C1 (BD Clontech). *ANXA4*-specific siRNA and negative control Stealth siRNA (Stealth™ RNAi) were purchased from Invitrogen. Cells were cultured in six-well plates or on coated cover slips for 24 h. Cells were then transiently transfected with pcDNA 3.1(+)/pEGFP-C1/ *ANXA4* (8 μg) or *ANXA4* siRNA (100 pmoles) using Lipofectamine 2000 (Invitrogen) according to the manufacturer's instructions. The efficiency of expression vector and siRNA transfection was determined by immunoblotting. After transfection for 48 h, the differential expression of proteins and genes was detected.

Antibodies

The mouse monoclonal antibodies used in this study were as follows: LAMP2 (ab25631) from Abcam; IgG_1 isotype (555746) from BD Biosciences; and α-tubulin (T5168) from Sigma. The rabbit polyclonal antibodies were as follows: The goat polyclonal antibody ANXA4 (sc-1930) was from Santa Cruz Biotechnology.

Live cell imaging

To determine ANXA4 localization after *H. pylori* infection, AGS or SC-M1 cells were plated on Lab-Tek™ chamber slide™ system (Nunc). After 24-h incubation, cells were transfected with pEGFP-C1/ *ANXA4* for 48 h prior to infection. *H. pylori* was resuspended in serum-free culture medium and stained with Hoechst 33258

(Sigma) for 1 h. The stained cells were centrifuged, washed twice, and then resuspended in fresh serum-free culture medium. Cultured cells were replaced with fresh serum-free culture medium (1 mL per well) and infected with *H. pylori* at a multiplicity of infection (MOI) of 150. Fluorescence images of living cells were captured by fluorescence microscopy (SC-M1: Zeiss Axiovert 200M (Zeiss); AGS: Nikon A1 confocal microscope (Nikon)). Images were processed using MetaMorph (Molecular Devices) software.

Flow cytometry

To measure intracellular $[Ca^{2+}]i$, cells were infected (MOI = 150) for 3 h, and both infected and non-infected cells were washed in Hanks' balanced salt solution (HBSS) buffer (137 mM NaCl, 5.33 mM KCl, 4.2 mM $NaHCO_3$, 0.44 mM KH_2PO_4, 0.34 mM Na_2HPO_4, 5.56 mM glucose; pH 7.3) and then loaded with 4 μM of the Ca^{2+} indicator, Fluo-3-AM /pluronic acid F-127, for 1 h at 37°C. Subsequently, cells were harvested using trypsin and then resuspended in HEPES buffer (137 mM NaCl, 5 mM KCl, 1 mM Na_2HPO_4, 5 mM glucose, 1 mM $CaCl_2$, 0.5 mM $MgCl_2$, 1 mg/ml BSA, and 10 mM HEPES, pH 7.4). Cells cultured in the presence of ionomycin (5 μM) were used as a positive control for increased Ca^{2+} concentration in the cytoplasm. To determine the cell surface expression of LAMP2, cells were resuspended in PBS and then fixed with 2% paraformaldehyde, centrifuged, washed in PBS, and blocked with 2% BSA/PBS for 15 minutes. The mouse anti-LAMP2 antibody and mouse IgG_1 isotype control were used at a dilution of 1:200 for 30 minutes at RT, following which the cells were washed and stained with anti-FITC secondary antibody for 30 minutes at RT. Cells were finally washed and fixed with 4% paraformaldehyde. Fluorescence intensity was determined using the FACSCalibur System (BD Biosciences), and the data were acquired by analyzing at least 10,000 cells from each sample.

References

1. Lai YP, Yang JC, Lin TZ, Wang JT, Lin JT (2003) CagA tyrosine phosphorylation in gastric epithelial cells caused by Helicobacter pylori in patients with gastric adenocarcinoma. Helicobacter 8: 235-243.
